# Supplementary material for: Diagnostic Potential of Endometrial Cancer DNA from Pipelle, Pap-Brush, and Swab Sampling
Source: Cancers (Basel). 2023 Jul 6;15(13):3522. doi: 10.3390/cancers15133522 (PMC10340240; doi:10.3390/cancers15133522)
Supplement: Supplementary file 1 [file cancers-15-03522-s001.zip › Supplementary Figures.pdf]

Supplementary Figures

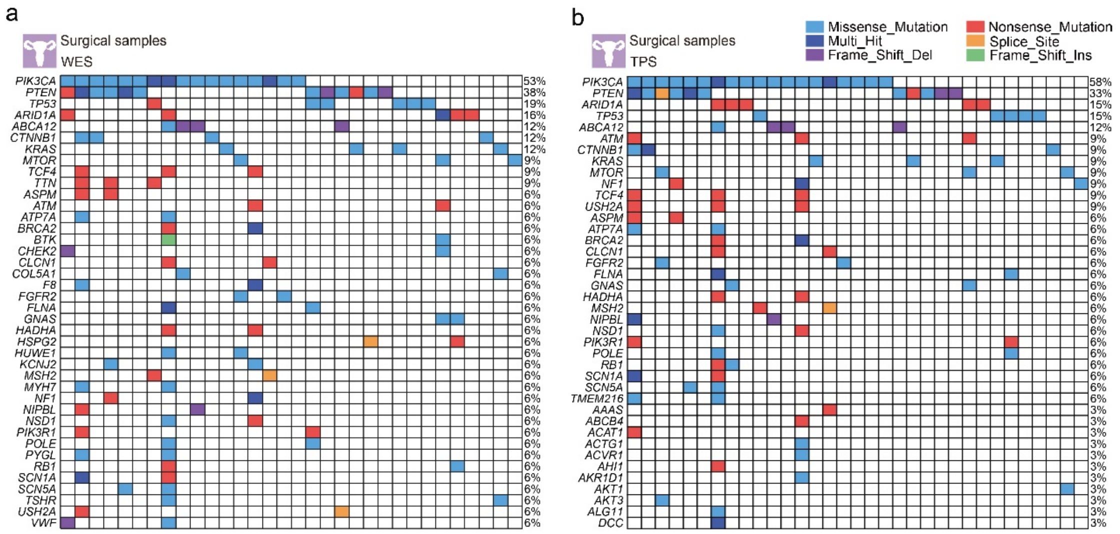

Figure S1. The landscape of frequently mutated genes of surgical samples from EC patients. (a) Top 40 mutated genes in 32 patients identified by WES. Right, the percentages of patients with mutations. Different colors correspond to different types of mutations. Variants annotated as Multhit_Hit are those genes that are mutated more than once in the same sample. (b) Top 40 mutated genes in 32 patients identified by TPS.

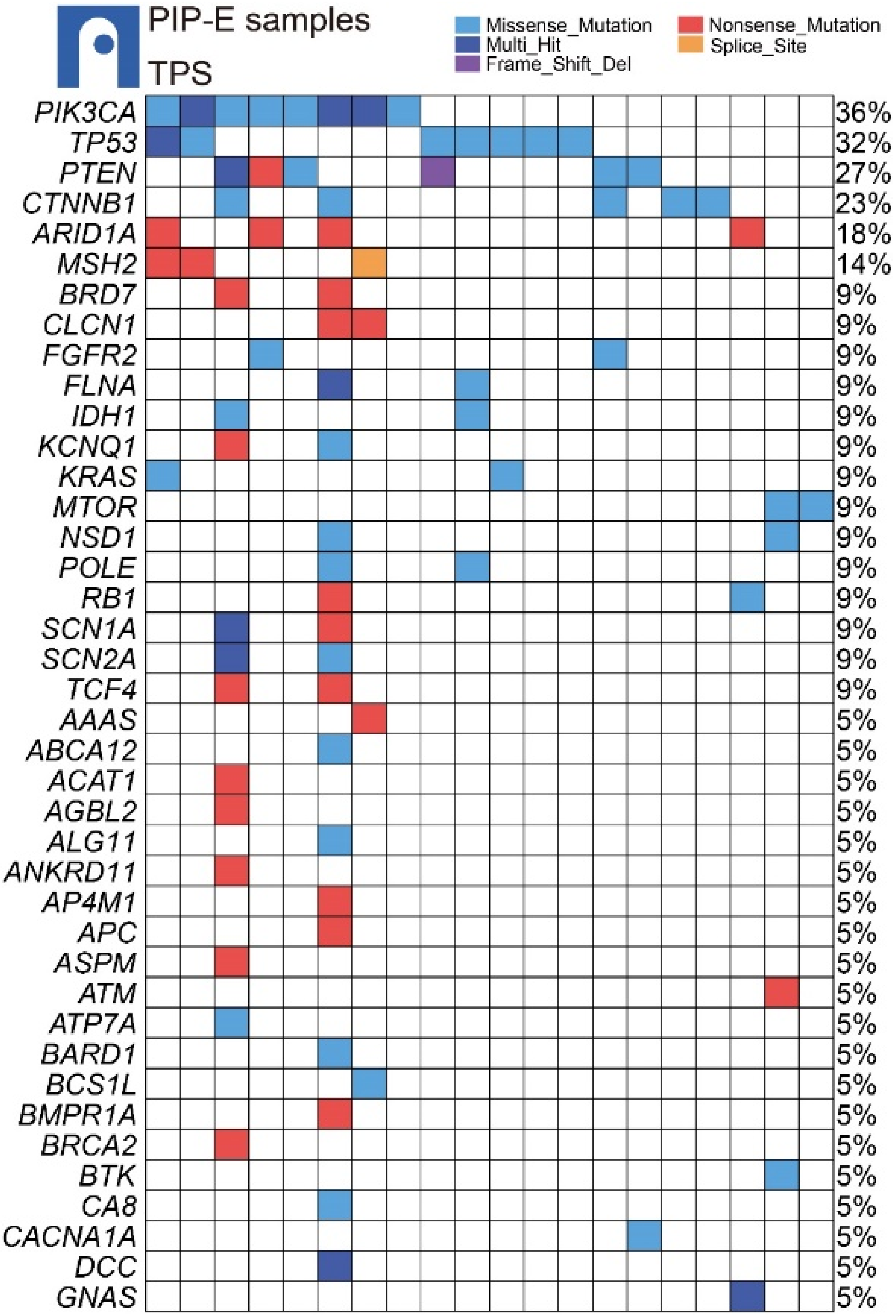

Figure S2. The landscape of frequently mutated genes of PIP-E samples from EC patients. Top 40 mutated genes in 22 patients identified by TPS. PIP-E: Pipelle endometrial.

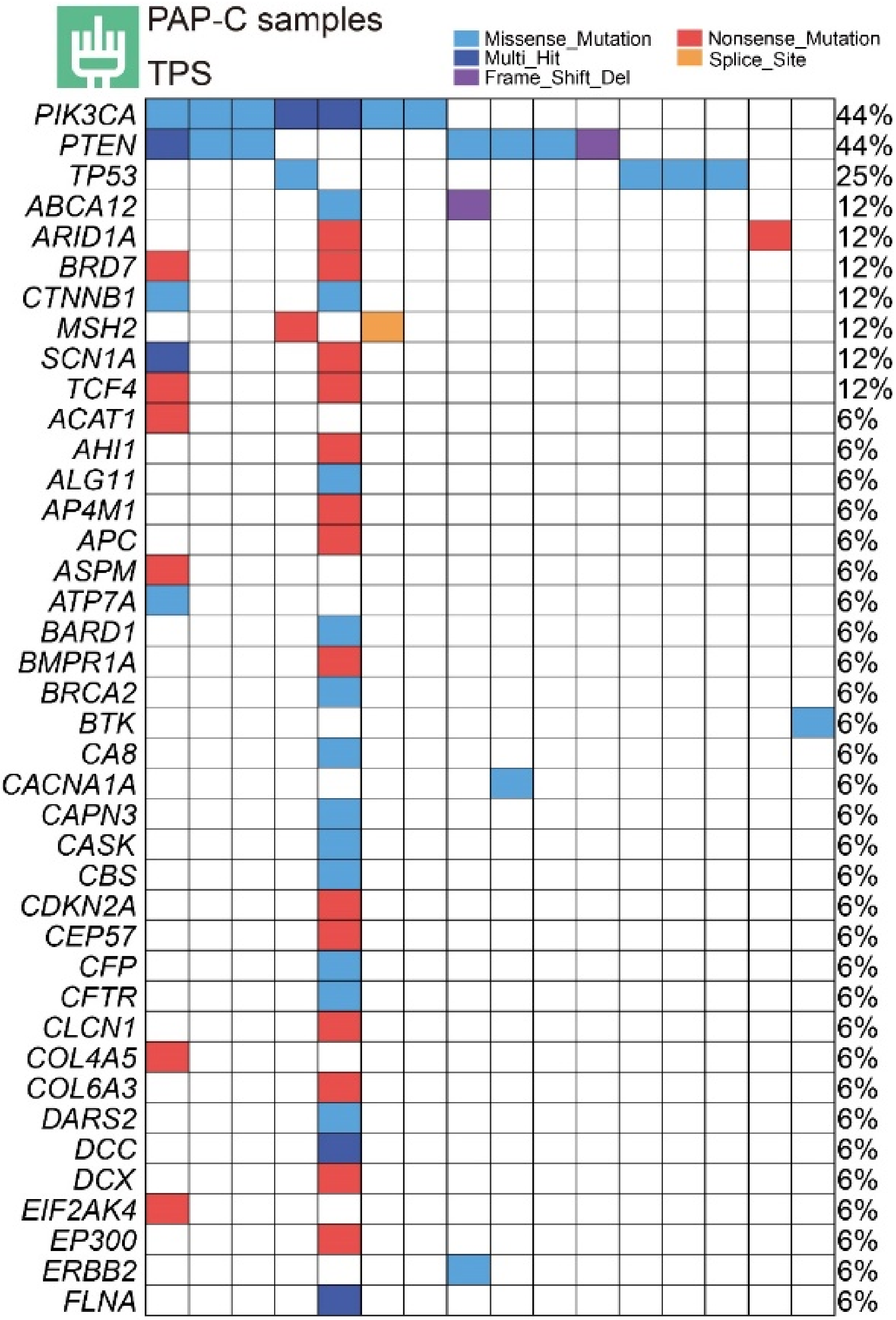

Figure S3. The landscape of frequently mutated genes of PAP-C samples from EC patients. Top 40 mutated genes in 16 patients identified by TPS. PAP-C: cervical Pap-brush.

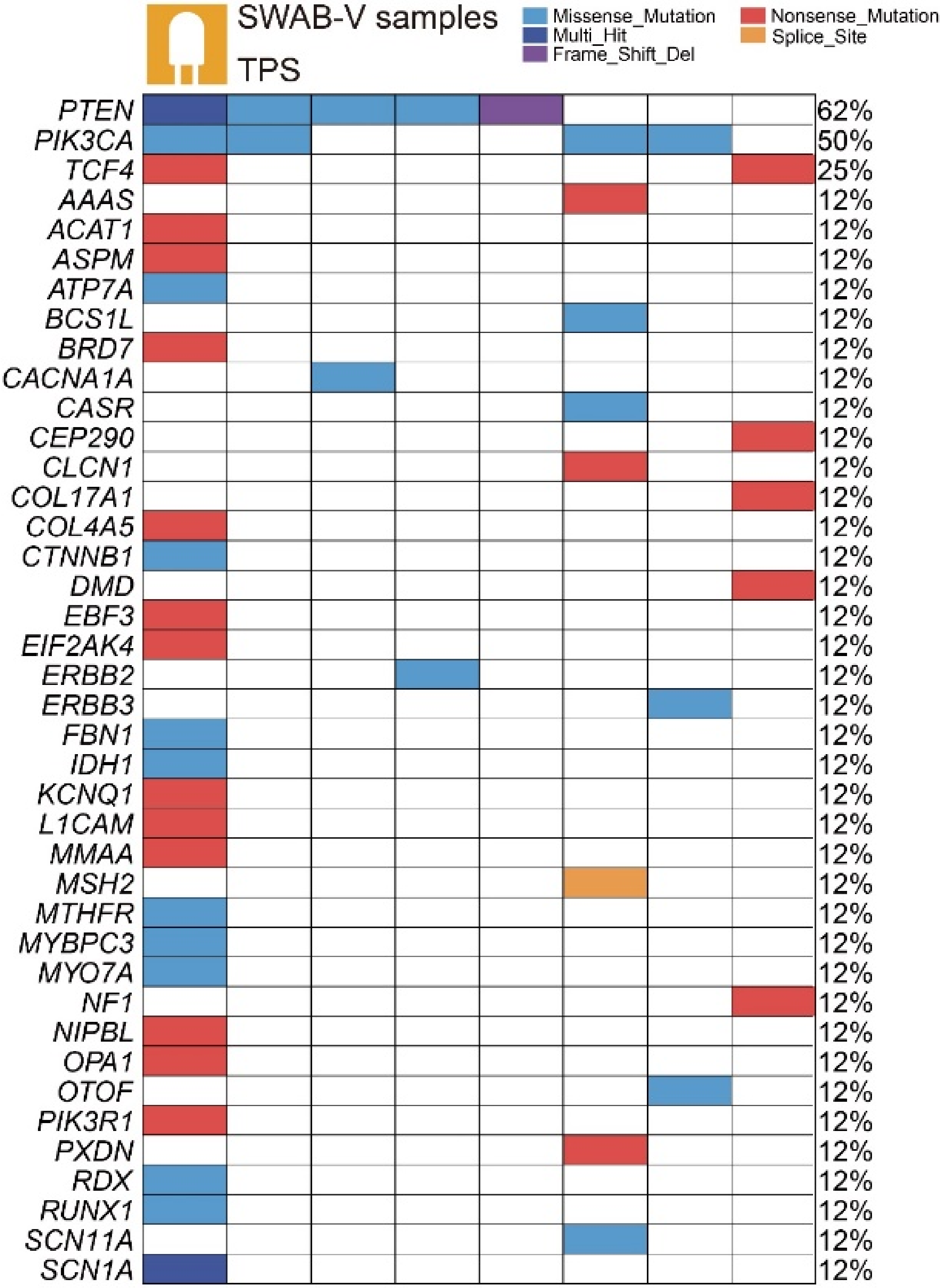

Figure S4. The landscape of frequently mutated genes of SWAB-V samples from EC patients. Top 40 mutated genes in 8 patients identified by TPS. SWAB-V: vaginal swab.

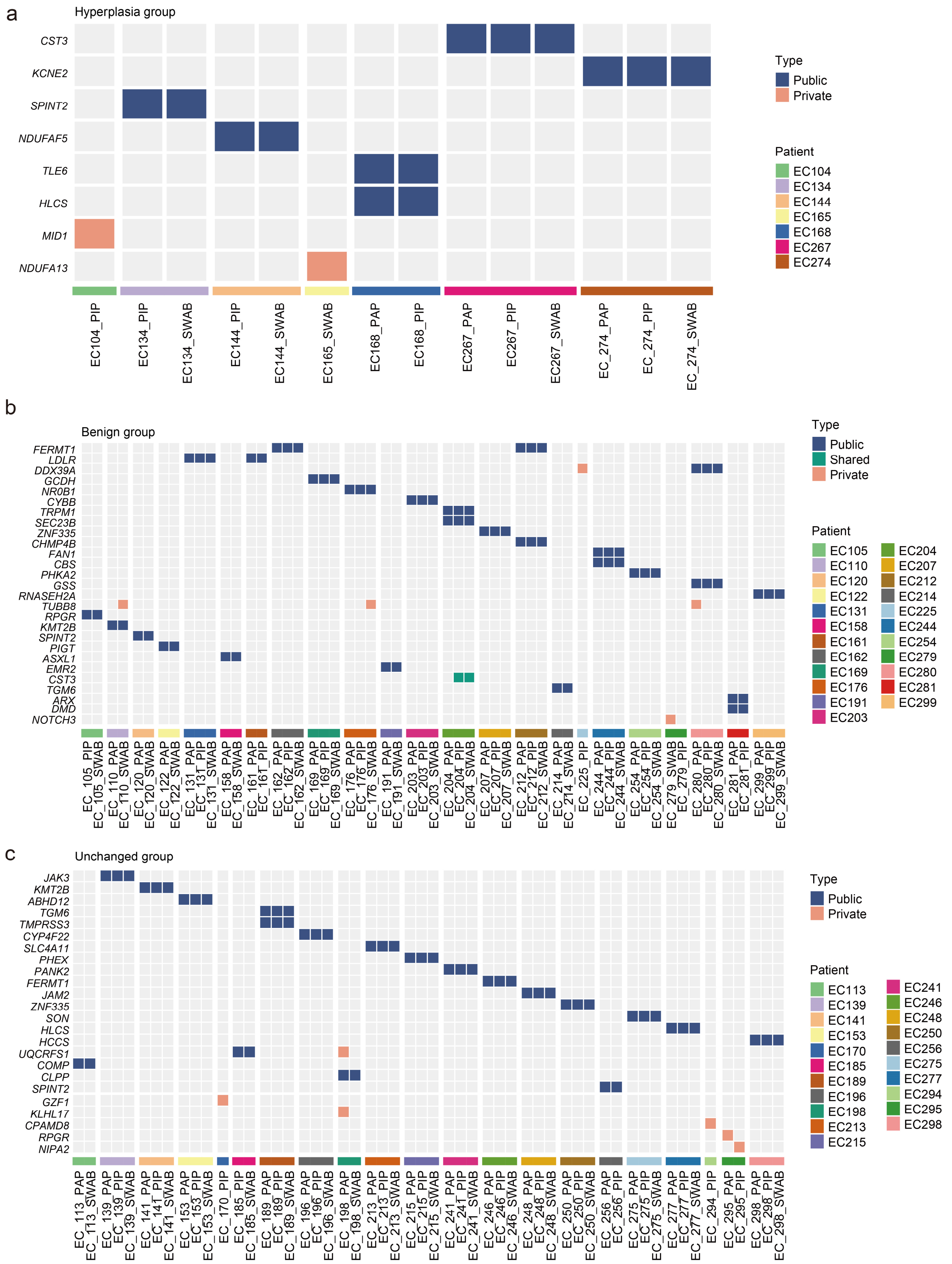

Figure S5. By-variant comparison of pathogenic mutations detected in endometrial, cervical, and/or vaginal samples from hyperplasia (a), benign (b), and unchanged (c) groups. Public indicates mutations existing in all samples, shared indicates mutations existing in part of all samples, and private indicates mutations existing in one type of sample. PIP-E, Pipelle endometrial biopsy; PAP-C, cervical Pap-brush sample; SWAB-V, vaginal specimen.
